# Video-assisted thoracic surgery of a caudal mediastinal paraoesophageal abscess in a cat with recurrent pyothorax

**DOI:** 10.1177/20551169261449116

**Published:** 2026-04-25

**Authors:** Mulan Zhong, Rachel Basa, Juan M Podadera, Mark B Krockenberger, Natalie Courtman, Mary F Thompson, Melanie Olive

**Affiliations:** Sydney School of Veterinary Science, Faculty of Science, The University of Sydney, NSW, Australia

**Keywords:** Pyothorax, paraoesophageal abscess, video-assisted surgery, thoracoscopy

## Abstract

**Case summary:**

A 6-year-old spayed female domestic shorthair cat was presented with acute respiratory distress and lethargy. Three months prior, the cat had been medically managed for a bacterial pyothorax. Initial stabilisation included oxygen supplementation, and a point-of-care ultrasound (POCUS) revealed bilateral pleural effusion. A thoracostomy tube was placed and pleural fluid was aseptically collected. Cytological evaluation of the pleural fluid was consistent with a septic neutrophilic exudate, with *Fusobacterium russii*, *Bacteriodes pyogenes* and *Porphyromonas gingivalis* isolated on anaerobic bacterial culture. A CT scan of the thorax revealed a caudal mediastinal paraoesophageal mass, with no gross mucosal abnormalities or foreign material apparent on oesophagoscopy. Lack of response to medical management promoted video-assisted thoracic surgery (VATS), with partial conversion to a thoracoscopic-assisted approach to improve visualisation. Histopathology of the mass was consistent with an abscess, and *F russii* and *B pyogenes* were isolated. No primary cause for the caudal mediastinal paraoesophageal abscess (CMPA) was identified. The cat was discharged with metronidazole after thoracostomy tube removal 4 days postoperatively. There was no recurrence of clinical signs and pleural effusion on POCUS at the 3-month follow-up.

**Relevance and novel information:**

CMPAs are rarely described in cats. This case outlines the clinical features, diagnostic investigations, treatment and outcome of a cat with a CMPA. The case reinforces the importance of repeat diagnostic imaging in cats with recurrent pyothorax and highlights the feasibility and challenges of VATS for CMPA removal, which has not been previously described in cats.

## Plain language summary

Video-assisted surgical removal of an abscess in the chest cavity of a cat with recurrent pyothorax

A 6-year-old, spayed female domestic shorthair cat was presented with difficulty breathing and lethargy. Three months prior, the cat was medically managed for an accumulation of fluid that contained bacteria and pus in the chest cavity (pyothorax). The cat was stabilised with oxygen supplementation, and ultrasound of the chest revealed an accumulation of fluid in the space around the lungs. A tube was placed into the chest to drain the fluid for therapeutic and diagnostic purposes. On drainage of the fluid from the chest, through placement of a thoracostomy tube, the fluid was found to be a recurrent pyothorax and bacteria was isolated on culture. A CT scan revealed a mass located towards the back and middle area of the chest, immediately next to the oesophagus (caudal mediastinal paraoesophageal region). There were no abnormalities of the inner lining of the oesophagus or foreign material apparent on endoscopy. As medical management had already been attempted previously, minimally invasive video-assisted surgery that required modification to improve visualisation was performed to remove the mass. The mass was consistent with an abscess, with bacteria isolated on culture. No cause of the caudal mediastinal paraoesophageal abscess was identified. The cat was discharged with metronidazole after chest tube removal 4 days after surgery. There was no recurrence of clinical signs or fluid in the space around the lungs on ultrasound at the 3-month follow-up.

## Introduction

Caudal mediastinal paraoesophageal abscesses (CMPAs) are rarely reported in the veterinary literature. The aetiology is poorly understood as the cause is often not identified.^1,2^ Described aetiologies include migrating or penetrating foreign bodies,^3,4^ oesophageal perforation^5,6^ and postoperative complications.^6^

Surgical intervention is primarily chosen for the management of CMPAs in dogs.^1,2,7,8^ Video-assisted thoracic surgery (VATS), a minimally invasive option, has been described in two cases.^2^ The use of VATS for CMPAs in cats has not been described. One cat was successfully managed with a right intercostal thoracotomy and the other euthanased while undergoing a median sternotomy.^3,9^ The euthanased cat had a grass awn identified on post-mortem examination.^3^ Successful medical management has been reported in cats, although one cat had a spontaneous rupture of the CMPA, leading to severe respiratory distress.^10,11^

This report describes a cat with a CMPA and recurrent pyothorax that was surgically managed utilising VATS. Surgery and antimicrobial therapy led to the resolution of clinical signs.

## Case description

A 6-year-old spayed female domestic shorthair cat was presented with respiratory distress and lethargy. The cat lived in a multi-cat household with outdoor access. Three months prior, the cat had been presented with similar clinical signs and medically managed with chest drainage and antimicrobials for presumed idiopathic pyothorax after a thoracic scan. Microbial culture of the pleural effusion identified *Fusobacterium russii* and *Bacteriodes pyogenes* and amoxycillin (23 mg/kg PO q12h for 4 weeks, Amoxycillin; Dechra) was prescribed. The cat presented 3 months later with a 24-h history of lethargy and dyspnoea. On presentation, the cat had respiratory distress and had dull lung sounds bilaterally. The cat was bright and alert with a normal body condition score (score 5/9, muscle condition score 1/4, body weight 4.52 kg), with no other significant abnormalities on physical examination. Oxygen supplementation was provided and a point-of-care-ultrasound (POCUS) revealed bilateral heterogeneous echogenic pleural effusion. A venous blood gas, haematology and serum biochemistry analysis were performed (see Table 1 in the supplementary material).

**Figure 1:**
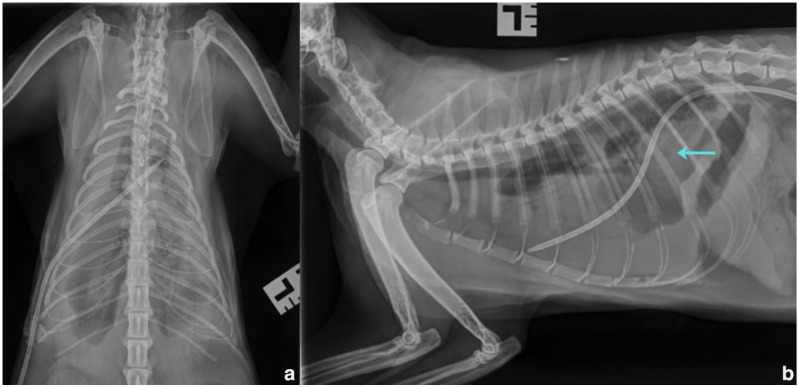
(a) Dorsoventral and (b) left lateral thoracic radiographs after chest drain placement. Soft tissue opacity in the caudal mediastinum (blue arrow) and bilateral pleural effusion evident

**Figure 2:**
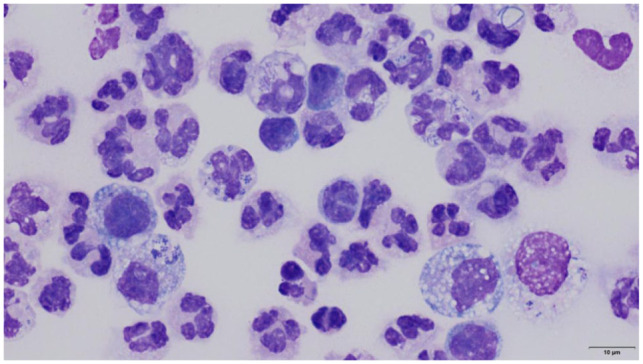
Cytology of the pleural fluid, cytospin smear stained with Wright’s Giemsa. The fluid is highly cellular and predominantly comprised of neutrophils and finely vacuolated macrophages, with rare lymphocytes. The neutrophils are moderately to markedly degenerate and frequent mixed intracellular bacteria are evident. Scale bar = 10 μm

A right-sided thoracostomy tube was placed using the Seldinger technique under sedation, with radiographic confirmation of placement (Figure 1).^12^ Beige milky fluid was retrieved (105 ml). Fluid cytology was consistent with a neutrophilic exudate and mixed bacterial infection (Figure 2). The fluid was submitted for aerobic and anaerobic culture.

Intravenous fluid therapy with lactated Ringer’s at a maintenance rate (11 ml/h), amoxycillin clavulanic acid (22 mg/kg IV q8h, Amoxyclav Juno; Juno) and methadone hydrochloride (0.2 mg/kg IV q4h ilium Methadone; Troy) were commenced. Pleural drainage followed by lavage with warm sterile 0.9% saline (30 ml repeated three times) was performed q4h. The cat remained in an oxygen cage (fraction of inspired oxygen at 50%).

**Figure 3:**
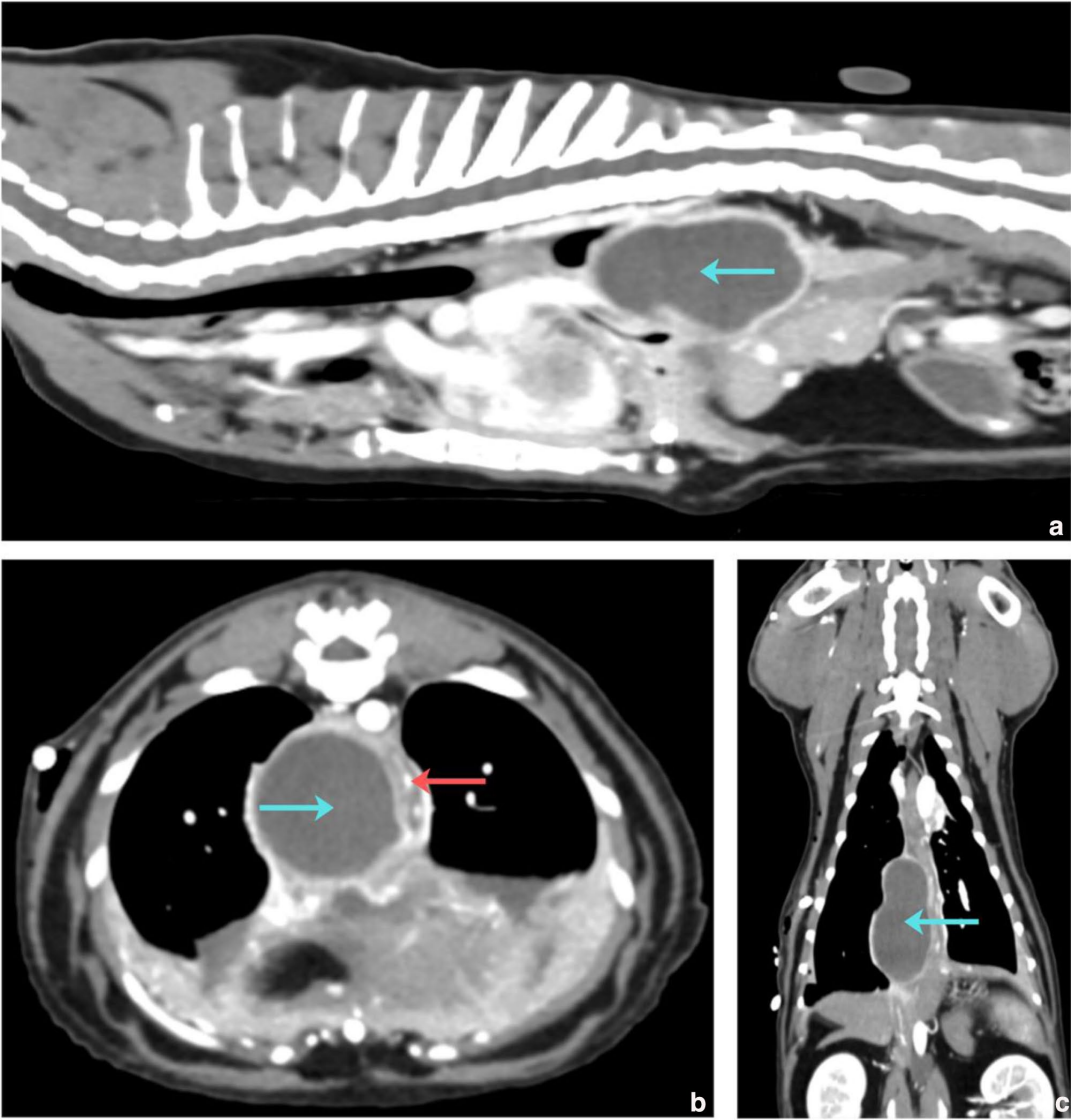
Post-contrast (a) sagittal, (b) transverse and (c) dorsal reconstructions from the CT scan of the thorax. There is a hypoattenuating ovoid area (4.6 × 2.5 × 2.5 cm) at the level of the paraoesophageal region of the right caudal mediastinum (blue arrows). The margins have strong contrast enhancement and there is mass effect with displacement of the oesophageal lumen (red arrow)

On the second day of hospitalisation, the cat’s respiratory rate and effort were within normal limits. The cat was premedicated with medetomidine hydrochloride (5 µg/kg IV, ilium Medetomidine; Troy), induced with alfaxalone (1 mg/kg slow IV, Alfaxan Multidose; Zoetis) titrated to effect and maintained on isoflurane inhalant for a thoracic CT scan. The scan revealed a wide hypoattenuating ovoid area with strong peripheral contrast-enhancing margins at the level of the caudal right paraoesophageal mediastinum (Figure 3). The dimensions of the mass were 4.6 × 2.5 × 2.5 cm. The findings were consistent with a cavitary paraoesophageal fluid-filled mass, suspected to be an abscess. The cat was weaned off oxygen supplementation. The thoracostomy tube was minimally productive (0.02–0.52 ml/kg/h).

After deliberation, the owner elected for surgical removal of the mass on day 4 of hospitalisation. The cat was premedicated with methadone hydrochloride (0.2 mg/kg IV, ilium Methadone; Troy) and medetomidine hydrochloride (2 µg/kg IV, ilium Medetomidine; Troy) induced with alfaxalone (1 mg/kg slow IV, Alfaxan Multidose; Zoetis) titrated to effect and maintained on isoflurane inhalant. Bupivacaine hydrochloride anhydrous (Bupivacaine Injection BP 0.5%; Pfizer) was administered to perform a serratus block (1.6 mg/kg) and intercostal block (0.55 mg/kg). A ketamine (10 µg/kg/min IV, Ketamine; Ceva) continuous rate infusion (CRI) was commenced for analgesia. Preoperative oesophagoscopy did not reveal any gross abnormalities of the oesophageal mucosa or foreign material and a percutaneous endoscopic gastrotomy tube was routinely placed because of hyporexia.

**Figure 4:**
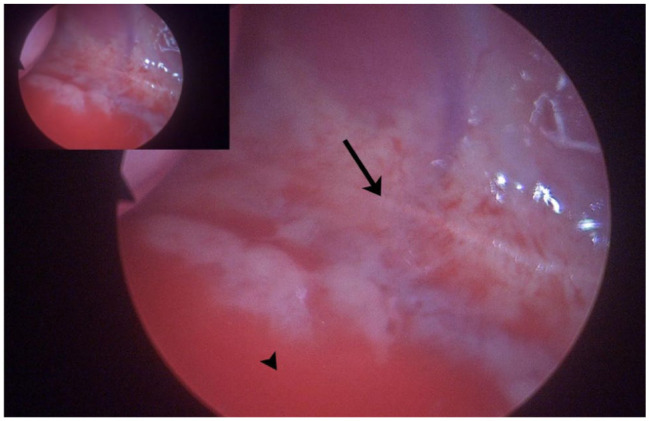
Thoracoscopic image of the caudal mediastinal paraesophageal abscess (black arrow) and right middle lung lobe (black arrowhead)

**Figure 5:**
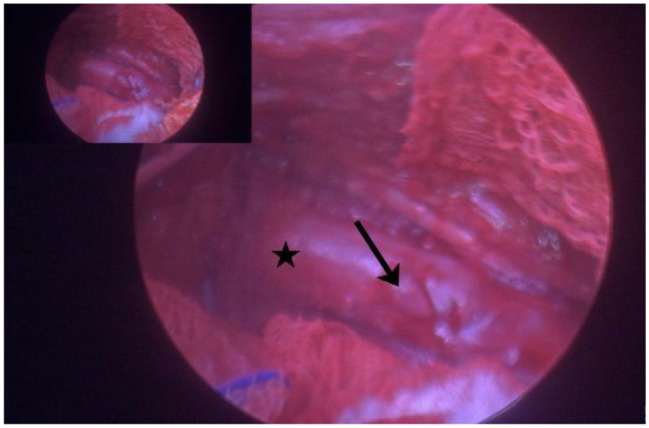
Video-assisted thoracic image after debridement of the caudal mediastinal paraoesophageal abscess (black arrow) allowing visualisation of the oesophagus (black star)

After oesophagoscopy, the cat was positioned in a 30° oblique left lateral recumbency and skin aseptically prepared. A 2 cm skin incision was made at the right mid- to dorsal- third to sixth intercostal space (ICS). Using straight mosquito haemostatic forceps, the subcutaneous tissues and intercostal muscles were bluntly dissected, and the pleura was similarly penetrated. A single incision facilitated insertion of a multichannelled port, through which three 6 mm cannulas were inserted. A 2.7 mm 0° short laparoscope was used to visualise internal thoracic structures. No foreign material was visualised within the thorax (Figure 4). Percutaneous aspiration of the abscess was unsuccessfully attempted. As a result of obscured visualisation and lack of available working space to safely dissect the CMPA, conversion to a thoracoscopic-assisted approach was elected. An eighth ICS approach was performed and an Alexis wound retractor placed. The CMPA was visualised, and purulent material (approximately 25 ml) was aspirated using a 20 G spinal needle and 12 ml syringe. The contents were submitted for microbial culture. A 5 mm laparoscopic Kelly grasping forceps and 5 mm laparoscopic Metzenbaum scissors were used to dissect and resect the majority of the CMPA. The oesophagus was visualised during the procedure and no perforations were evident (Figure 5). A segment of the CMPA wall was submitted for histopathology and microbial culture. The thorax was thoroughly lavaged with sterile saline solution. Under direct visualisation, the thoracostomy tube was replaced. All incisions were closed routinely. Recovery from anaesthesia was uneventful.

The cat was administered the following medications postoperatively: amoxycillin clavulanic acid (13.8 mg/kg PO q8h, Amoxyclav; Dechra), enrofloxacin (5 mg/kg PO q24h, Baytril; Elanco), fentanyl (3–5 µg/kg/min IV, Fentanyl GH; DHL Generic Health) CRI, ketamine (3–5 µg/kg/min IV, Ketamine; Ceva) CRI, meloxicam (0.2 mg/kg SC once, ilium Meloxicam; Troy) and bupivacaine hydrochloride anhydrous (1.5 mg/kg via the thoracostomy tube q5h, Bupivacaine Injection BP 0.5%; Pfizer). The thoracostomy tube was aspirated and lavaged q8h.

The cat was hospitalised for 4 days postoperatively and the chest drain was removed 3 days postoperatively because of minimal fluid production (0.03–0.19 ml/kg/h). The fluid was clear and slightly serosanguineous. Anaerobic culture of the pleural effusion identified *F russii*, *B pyogenes* and *Porphyromonas gingivalis*; aerobic culture was negative after 7 days. Susceptibility testing for obligate anaerobes could not be performed at the laboratory involved. Antimicrobial therapy was changed to metronidazole (11 mg/kg PO q24h, Metrogyl; Arrow Pharmaceuticals) for 14 days to target anaerobes. The gastrotomy tube was not utilised as the cat’s appetite improved.

**Figure 6:**
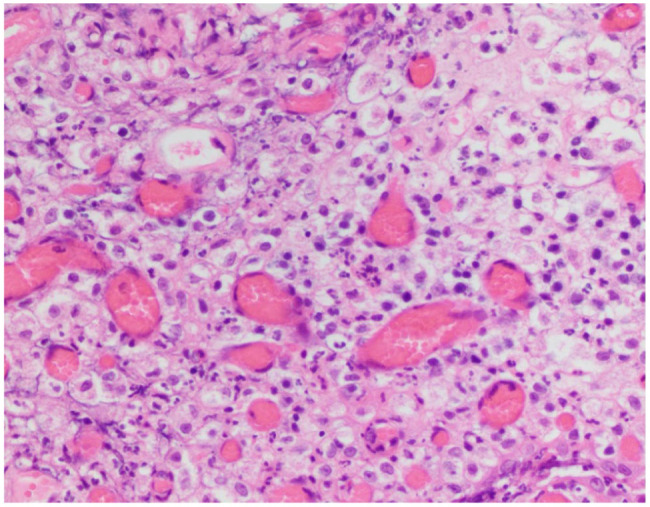
Histological section of the paraoesophageal mass, haematoxylin and eosin stain (200× magnification). Among the numerous engorged small vessels is an inflammatory infiltrate dominated by large numbers of degenerate and non-degenerate neutrophils. The findings are consistent with marked granulation tissue with suppurative inflammation, consistent with an abscess wall. No pathogens or foreign material were identified

Histopathological examination of specimens from the paraoesophageal mass revealed variable numbers of remnant smooth muscle fibres interspersed with fibroplasia and numerous small vessels lined by plump endothelium. Numerous neutrophils and lower numbers of macrophages were scattered throughout the specimen and multifocal aggregates of small lymphocytes were present. Overall interpretation was that the specimens represented maturing granulation tissue and chronic active inflammation as would be expected in an abscess wall (Figure 6). No infectious agents were identified on histopathology. Anaerobic culture of the tissue identified *B pyogenes* and *F russii*; aerobic culture was negative after 7 days.

The cat was rechecked weekly after discharge for 1 month, and the owner reported no concerns with her breathing or activity level. The gastrotomy tube was removed 3 weeks postoperatively. A recheck at 3 months postoperatively revealed no significant abnormalities, and a POCUS detected no significant abnormalities or pleural effusion.

## Discussion

This report documents a cat with a CMPA managed utilising VATS that required partial conversion from a total thoracoscopic procedure to a thoracoscopic-assisted approach to facilitate safer dissection of the abscess. The case highlights the benefits and challenges of VATS as a minimally invasive surgical option for CMPAs in cats. To the authors’ knowledge, this is the first report of VATS in a cat with a CMPA.

Surgical intervention for the CMPA was chosen as medical management had already been attempted during the first episode of pyothorax, albeit in the absence of a CMPA, and considering documented positive outcomes in dogs and cats.^1,2,7,9^ Traditional open thoracotomy has been more frequently reported.^1,7,9^ VATS is a minimally invasive alternative with reported advantages, including a shorter hospitalisation, lower postoperative complications and reduced postoperative pain.^13^ The cat in this report was hospitalised for 4 days postoperatively, with the thoracostomy tube removed on postoperative day 3. The postoperative outcome is consistent with dogs managed with VATS that were hospitalised 3–4 days postoperatively, with thoracostomy tubes removed 1 day before discharge.^2^ This is in contrast to open thoracotomy management of a cat with CMPA that required pleural drainage and lavage 2 weeks postoperatively and thoracostomy drain removal on day 29 after discharge.^9^ Further studies are recommended to compare the surgical outcomes of VATS with open thoracotomies in dogs and cats.

A partial conversion to a thoracoscopic-assisted approach was required to improve visualisation and create adequate working space, challenges particularly appreciated in cats and smaller dogs.^14^ One-lung ventilation and low-pressure carbon dioxide insufflation are strategies that can be used concurrently with VATS to enhance working space.^15^ These techniques can cause cardiorespiratory compromise, although this was minimal in a study of clinically healthy cats that experienced no detrimental decline in oxygen delivery despite significant decreases in PaO_2_ and SpO_2_.^15^ Another consideration is the possible increased anaesthetic time required to create one-lung ventilation.^15^ The development of newer techniques, including fluoroscopy-assisted endobronchial intubation, can minimise this drawback.^16^ In a report of two dogs undergoing VATS for CMPAs, one dog required partial conversion similarly because of inadequate working space and limited visualisation.^2^ The other dog had a complete thoracoscopic procedure, but was mildly hypoxic during recovery, suspected to be secondary to persistent atelectasis.^2^ A complete thoracoscopic procedure for cats with CMPAs is likely feasible with concurrent strategies to reduce the challenges of visualisation and working space.

The cause of the CMPA in the current case was not determined. The isolation of bacterial species consistent with oropharyngeal flora supports oesophageal perforation, aspiration or haematogenous spread from a distant wound.^17^ As the abscess was adjacent to the oesophagus, a previous oesophageal perforation remains a possibility. Foreign body involvement by direct inoculation of the oesophagus or migration is plausible. Oesophagoscopy performed preoperatively did not reveal mucosal abnormalities, but this does not exclude a resolved oesophageal lesion, especially considering that the first pyothorax was documented 3 months prior. The use of enrofloxacin is a limitation from an antimicrobial stewardship perspective, and because of the risk of retinal degeneration in cats.^18^

## Conclusions

This case highlights the feasibility and challenges of VATS as a minimally invasive surgical option for cats with CMPAs. Further developments in performing one-lung ventilation concurrently and investigations into outcomes of cats undergoing VATS compared with open thoracotomies are required to better inform decision-making. The case reinforces the necessity of repeat thoracic imaging in cats with recurrent pyothorax.

## Supplemental Material

Table 1 Venous blood gas, haematology and serum biochemistry results performed on admission.
