# Supplementary material for: Video-assisted thoracic surgery of a caudal mediastinal paraoesophageal abscess in a cat with recurrent pyothorax
Source: JFMS Open Rep. 2026 Apr 25;12(1):20551169261449116. doi: 10.1177/20551169261449116 (PMC13172676; doi:10.1177/20551169261449116)
Supplement: Table 1 [file sj-docx-1-jor-10.1177_20551169261449116.docx]

**Table 1** Venous blood gas, haematology, and serum biochemistry results performed on admission

| Venous blood gas | | Haematology | | Serum biochemistry | |
| --- | --- | --- | --- | --- | --- |
| pH | 7.359 (7.31-7.46) | Haematocrit (L/L) | 0.31 (0.30-0.45) | **Creatine Kinase (U/L)** | **957 (66-447)** |
| pCO_2_ (mmHg) | 38.1 (38-47) | Total Protein (g/L) | 74 (68-91) | ALP (U/L) | 26 (13-54) |
| Sodium (mmol/L) | 155 (147-156) | **Platelet Count (x10^9^/L)** | **293 (300-700)** | ALT (U/L) | 18 (4-64) |
| Potassium (mmol/L) | 4.0 (4.0-4.5) | Leukocytes (x10^9^/L) | 10.3 (2-14) | **AST (U/L)** | **49 (0-26)** |
| Chloride (mmol/L) | 121 (115-130) | Segmented Neutrophils (x10^9^/L) | 4.84 (1.88-10.26) | GGT (U/L) | 3 (1.0-5.0) |
| Ionised calcium (mmol/L) | 1.24 (1.1-1.4) | **Band Neutrophils (x10^9^/L)** | **2.16 (0-0.42)** | Total Protein (g/L) | 75 (63-88) |
| Lactate (mmol/L) | **4.1 (<2.5)** | Lymphocytes (x10^9^/L) | 1.75 (0.5-8.1) | **Albumin (g/L)** | **24 (27-40)** |
| Glucose (mmol/L) | 5.7 (3.3-6.4) | Monocytes (x10^9^/L) | 0.62 (0-0.62) | Globulins (g/L) | 51 (32-52) |
| Bicarbonate (mmol/L) | **21.5 (17-21)** | Eosinophils (x10^9^/L) | 0.72 (0.04-1.90) | **Total bilirubin (umol/L)** | **4.7 (0.2-4.2)** |
| SBE (mmol/L) | -4 (-4-+4) | **Metamyelocytes (x10^9^/L)** | **0.21 (0-0)** | Creatinine (umol/L) | 74 (68-167) |
| Anion Gap (mmol/L) | **16.9 (17-31)** |  |  | Urea (mmol/L) | 7.2 (4.3-11.7) |
|  |  |  |  | **Total T4 (nmol/L)** | **16.4 (20-44)** |

pCO_2_ = partial pressure of carbon dioxide; SBE = standard base excess; ALP = alkaline phosphatase; ALT = alanine aminotransferase; AST = aspartate aminotransferase; GGT = gamma-glutamyl transferase.
